# Genetic Basis of Blood-Based Traits and Their Relationship With Performance and Environment in Beef Cattle at Weaning

**DOI:** 10.3389/fgene.2020.00717

**Published:** 2020-07-03

**Authors:** Josue Chinchilla-Vargas, Luke M. Kramer, John D. Tucker, Donald S. Hubbell, Jeremy G. Powell, Toby D. Lester, Elizabeth A. Backes, Karen Anschutz, Jared E. Decker, Kenneth J. Stalder, Max F. Rothschild, James E. Koltes

**Affiliations:** ^1^Department of Animal Science, Iowa State University, Ames, IA, United States; ^2^Division of Agriculture, Livestock and Forestry Research Station, Batesville, AR, United States; ^3^Department of Animal Science, University of Arkansas, Fayetteville, AR, United States; ^4^Division of Animal Science, University of Missouri, Columbia, MO, United States

**Keywords:** beef cattle, hemoglobin, weaning, heritability, GWAS - genome-wide association study, weaning weight, white blood cell count, complete blood cell (CBC) count

## Abstract

The objectives of this study were to explore the usefulness of blood-based traits as indicators of health and performance in beef cattle at weaning and identify the genetic basis underlying the different blood parameters obtained from complete blood counts (CBCs). Disease costs represent one of the main factors determining profitability in animal production. Previous research has observed associations between blood cell counts and an animal’s health status in some species. CBC were recorded from approximately 570 Angus based, crossbred beef calves at weaning born between 2015 and 2016 and raised on toxic or novel tall fescue. The calves (*N* = ∼600) were genotyped at a density of 50k SNPs and the genotypes (*N* = 1160) were imputed to a density of 270k SNPs. Genetic parameters were estimated for 15 blood and 4 production. Finally, with the objective of identifying the genetic basis underlying the different blood-based traits, genome-wide association studies (GWAS) were performed for all traits. Heritability estimates ranged from 0.11 to 0.60, and generally weak phenotypic correlations and strong genetic correlations were observed among blood-based traits only. Genome-wide association study identified ninety-one 1-Mb windows that accounted for 0.5% or more of the estimated genetic variance for at least 1 trait with 21 windows overlapping across two or more traits (explaining more than 0.5% of estimated genetic variance for two or more traits). Five candidate genes have been identified in the most interesting overlapping regions related to blood-based traits. Overall, this study represents one of the first efforts represented in scientific literature to identify the genetic basis of blood cell traits in beef cattle. The results presented in this study allow us to conclude that: (1) blood-based traits have weak phenotypic correlations but strong genetic correlations among themselves. (2) Blood-based traits have moderate to high heritability. (3) There is evidence of an important overlap of genetic control among similar blood-based traits which will allow for their use in improvement programs in beef cattle.

## Introduction

Expenses associated with disease and feed are two of the main drivers for cost of production in livestock operations and show a direct relationship where disease impacts feed intake (Irsik et al., 2006; Leach et al., 2013). There is limited information in scientific literature regarding genetic parameters for blood-based traits in livestock and the majority of scientific literature regarding this topic in beef cattle dates back to the second half of the 20th century.

With the greater use of molecular genetics by seedstock and commercial beef producers, animal breeding has experienced a paradigm shift (Meuwissen et al., 2016), with a larger number of traits and information being used to increase the accuracy to identify the best animals across a range of environments and production settings. Therefore, blood-based traits and other “forgotten” traits should be evaluated again using the methods currently available to better understand their usefulness in modern animal production.

In beef cattle, bovine respiratory disease (BRD) is among the most economically important traits in production (Snowder et al., 2007; Schneider et al., 2009). Infection can result in morbidity, mortality, and reduced average daily gain, which ultimately translates into reduced product quality and an overall reduced system productivity (Griffin, 1997; Irsik et al., 2006; Fulton, 2009; Leach et al., 2013). With blood samples being relatively easy to obtain when handling animals for other procedures and the intrinsic presence of white blood cells in peripheral blood, blood counts are an objective representation of innate and adaptive immunity of the animals (Leach et al., 2013). In this regard, Leach et al. (2013) looked at the genetic correlation between immune response to Bovine Respiratory Disease (BRD) vaccine and the incidence of the disease and average daily gain (ADG). They reported that blood-based traits related to immunity such as neutrophils (NE), lymphocytes (LY), eosinophils (EO) and basophils (BA) change significantly over time depending on the vaccination status of the animal (before or after a vaccination booster is applied). The research also showed significant correlations between the blood-based traits and ADG.

Previous efforts to identify genetic parameters for blood-based traits in beef cattle include those by Rowlands (Rowlands et al., 1977, 1983) and Richardson (Richardson et al., 1996). Rowland estimated a heritability of 0.55 ± 0.18 and a genetic correlation of -0.46 with growth rate for hemoglobin concentration in blood (Rowlands et al., 1983). While Richardson also calculated a repeatability ranging from 0.43 to 0.95 for various blood-based traits. More recently, Leach et al. (2013) found genetic correlations ranging from -0.48 to 0.86 between blood-based traits related to immunity.

Blood-based traits and their genetic basis have been given more attention in swine, perhaps because of the translational potential to humans that swine possess. Clapperton et al. (2008) compared heritability and genetic and phenotypic correlations of blood-based traits between herds with high and low health status at 30 kg and 90 kg of live weight. They found that heritability for white blood cell traits can vary greatly between herds exposed to different environments with the heritability for number of white blood cells changing from 0.06 ± 0.11 in high health herds to 0.37 ± 0.16 in low health herds. Additionally, they found mostly strong negative correlations between traits related to white blood cells and ADG ranging from 0.03 to -0.62. Evidence for heritability and moderate to strong genetic correlations of Blood-based traits to growth traits could be helpful in identifying and selecting animals with more robust growth under stressful environments. Robust growth is defined as the ability, in the face of environmental constraints, to carry on doing the various things that the animal needs to do to express its full genetic potential through rapid growth and weight gain (Friggens et al., 2017).

More recently, Flori et al. (2011) calculated heritability for total white blood cells (WBC) 0.73 ± 0.20 and 0.80 ± 0.21 for EO in swine. Mpetile et al. (2015) compared peripheral blood profiles from complete blood counts (CBCs) between lines of pigs selected for high and low residual feed intake (RFI). They found no significant correlations between RFI and the blood-based traits studied. Heritability estimates ranged from 0.04 for mean corpuscular hemoglobin concentration (MCHC) to 0.62 for red blood cells count (RBC).

In a very similar study to the one presented in our report, although on swine, Bovo et al. (2019) performed a genome-wide association analyses (GWAS) for 15 hematological traits and 15 clinical-biochemical traits finding 52 quantitative trait loci (QTL) associated with 29 of the 30 traits investigated. They also estimated genomic variance parameters and (SE) for blood-based traits and observed heritabilities ranging from 0.14 (0.06) for EO to 0.40 (0.06) for mean corpuscular hemoglobin (MCH).

Overall, previous studies have indicated that blood-based traits may be useful as indicators for performance and health in intensive production settings. The objectives of this study were to explore the usefulness of blood-based traits as performance and health in beef cattle at weaning and identify the genetic basis underlying the different blood parameters obtained from complete blood counts (CBCs).

## Materials and Methods

### Data Description

Complete blood count (CBC)s were recorded from 570 crossbred cattle (Angus background crossed with Hereford, Charolais, Sim-Angus, Brangus) using blood samples collected at weaning during 2015 and 2016 at three research farms with similar management techniques at the University of Arkansas in Fayetteville and Batesville, AR. Animals were handled in accordance with the regulations of the University of Arkansas Institute for Animal Care and Use Committee (IACUC), under protocol number 16037. Blood samples were collected at weaning via jugular vein puncture into an EDTA blood tube and analyzed in a Hemavet HV 950 multispecies hematology system (HEMAVET, 2011). In addition, birth weight, weaning weight and age at weaning were collected for all animals. Additionally, average daily gain and adjusted weaning weight at 205 days were calculated. The number of records from each farm is shown in Table 1, along with their respective year and calving season. Table 2 shows the traits included in the analyses along with their respective abbreviations and units. Animals raised at Savoy farm were raised on toxic fescue until weaning, while the majority of animals raised at Batesville and North farms were moved to novel fescue upon calving and were kept there until weaning.

**TABLE 1:** Distribution of animals by farm, year and calving season.

**Farm**	**Year**	**Calving Season**	**Number of records**
Savoy	2015	Spring	-
		Fall	205
	2016	Spring	76
		Fall	-
Batesville	2015	Spring	-
		Fall	72
	2016	Spring	38
		Fall	157
North	2015	Spring	-
		Fall	-
	2016	Spring	22
		Fall	-

**TABLE 2:** Description of traits analyzed.

**Trait**	**Abbreviation**	**Unit**
Hemoglobin content	HB	g/dL
Hematocrit percentage	HCT	%
Mean corpuscular hemoglobin	MCH	Pg
Mean corpuscular volume	MCV	fL
Mean corpuscular hemoglobin concentration	MCHC	g/dL
Red blood cells	RBC	M/uL
Red blood cell distribution width	RDW	%
Basophils	BA	K/uL
Basophils(logarithm)	BAlog	Log
Eosinophils	EO	K/uL
Lymphocytes	LY	K/uL
Monocytes	MO	K/uL
Neutrophils	NE	K/uL
White blood cells	WBC	K/uL
Mean platelet volume	MPV	fL
Platelets	PLT	K/uL
Birth weight	BW	lbs
Weaning weight	WW	lbs
Adjusted weaning weight^1^	adjWW	lbs
Average daily gain	ADG	lbs

^1^ Calculated as: ((*weaning weight*−*birth weight*)/*days at weaning*)*205.

Blood samples were collected for DNA isolation following previously described methods (Sambrook, 2001), and subsequently for genotyping. Animals were genotyped using the GeneSeek Bovine GGP50 SNP chip or the GGP F250 SNP chip from GeneSeek (NEOGEN, 2016). Approximately 1100 animals related to the CBC-phenotyped individuals, including their parents were genotyped with the GGP F250 chip and their genotypes were used for imputation purposes. Genotype positions for all SNP were updated to coordinates of the ARS1.2 bovine reference genome^1^.

### Imputation

A total of 501 animals with CBC records were genotyped at a density of approximately 50 k markers while 1160 animals from the same population, including the parental generations were genotyped at a density of approximately 250k markers. FImpute version 2.2 (Sargolzaei et al., 2014) was used to impute all genotypes to an approximate density of 270k markers. The resulting genotypes where used for further analyses. Imputation accuracy was not measured for the present project but previous experiences with similar projects have shown accuracies ranging between 90% and 95%.

### Population Structure

The population analyzed was divided in six contemporary groups defined by the combination of farm of origin, year of calving and calving season as shown in Table 1. To visualize population structure, a principal component analysis (PCA) was performed (results not shown). For this purpose, genotypes of registered purebred Hereford, Black Angus, Red Angus, Gelbvieh, Limousine, Simmental and Shorthorn animals were used as references to quantify the genomic similarity between the individuals used in this study.

### Statistical Analyses

#### Frequentist Approach to Estimate Phenotypic, Genetic and Genomic Parameters

Phenotypic correlations between traits were estimated using the method of moments after adjusting the data for fixed effects that included contemporary group and sex. Genotypic correlations between traits and narrow sense heritability (h^2^) for each trait were calculated in ASReml 3.0 (Mary et al., 2009) using an animal model with a genomic relationship matrix and fixed effects for contemporary group (***C*_*i*_**), sex of the animal (***S*_*j*_**), the genetic random effect of the animal (***D*_*k*_**) and a covariate for weaning weight (***W***):

(1) P1Pi⁢j⁢k2=i⁢j⁢kμ+C+iS+jD+kW+ϵi⁢j⁢k

For genetic correlations, a bivariate model was used while for heritability a univariate model with the same effects was implemented (***P1*** = phenotype 1, ***P2*** = phenotype 2).

#### Bayesian Approach to Estimate Genomic Parameters and Identify Genome-Wide Marker Associations

Narrow sense heritability (h^2^) for each trait was estimated through a Bayesian analysis in GenSel4.0 (Fernando and Garrick, 2008) utilizing Bayes C (Habier et al., 2011). For these analyses, only markers with a minor allele frequency (MAF) larger or equal to 0.02 were used. With this filter, it was guaranteed that at least 10 animals with the minor allele were included in the analyses. A large proportion of markers had MAF lower than 0.02. After filtering, approximately 100,000 markers were retained. Additionally, the Pi value was set to 0.9877 in order to fit the random effect of approximately 250 markers to the model per iteration. Each chain consisted of 75000 iterations with a burn of the first 5000 (i.e., the first 5000 samples were discarded). The same parameters were used to perform a genome-wide association study (GWAS) with the objective of exploring the genetic basis of blood-based traits. For this purpose Bayes B (Stephens and Balding, 2009) was used as it shrinks the genetic effects, with a larger shrinking factor on windows that show smaller genetic effects (Fernando and Garrick, 2013). For GWAS purposes, variance was examined for fixed windows of 1 Megabase (Mb) genomic segments. When estimating heritability for each trait and performing a GWAS, the model used was the same one used to estimate genomic heritability, composed of fixed effects contemporary group (***CG*_*i*_**), sex of the animal (***S*_*j*_**), a covariate for weaning weight (***WW***) and the random effects of the markers (***M*_*l*_**) fitted during each iteration:

(2) P=i⁢j⁢k⁢lμ+CG+iS+jM+lWW+ϵ

#### Gene Ontology and Identification of Candidate Genes

To be considered significant, windows had to explain at least 0.5% of the estimated genetic variance. This threshold was assigned with the rationale that there is little literature referring to blood-based traits and it is not known if these traits are affected by a few genes with large effects or multiple genes with small effects. Genes located in each significant genomic window were identified with Ensembl Biomart^2^ by choosing the “cow genome” option with the ARS1.2 bovine genome version. Given the small number of annotated genes in most of the windows found significant, traits were grouped in four broad categories to increase the probability of detecting enrichment for specific categories: red blood cell traits, white blood cell traits, platelet traits and growth traits (i.e., MCHC and RBC were grouped under red blood cell traits). This, under the rationale that traits related to the same type of cells should share molecular basis (Iwasaki and Akashi, 2007). Once the list of genes located in all significant windows for a category of traits was obtained, an ontology enrichment test was performed through Princeton University’s Lewis-Sigler Institute for Integrative Genomics GO:TermFinder (Boyle et al., 2004). For a term enrichment to be considered significant false discovery rate (FDR) had to be less than 5%. Given that the annotation of the human genome is far more complete when compared to the annotation on the cow’s genome, all gene ontology terms were performed using the human genome as a reference. This approach, as discussed by Band et al. (2000) is a very useful tool to discover genes of agricultural importance.

Finally, the genes located in windows that explained ≥ 0.5% of the estimated genetic variance in two or more traits were investigated with the objective of identifying possible candidate genes to be associated to the estimated genetic variance explained by each window. Genes were considered candidates when scientific literature linking the genes to physiological processes related to blood-based traits.

## Results

### Population Structure

There was a wide range of crossbred animals genotyped for this study. However, it should be noted that there is a small set of animals with a heavy Black Angus background. Given the high level of heterogeneity shown by PCA (Proportion of variance explained: PC1 = 0.047, PC2 = 0.025, PC3 = 0.014), it was decided to ignore the animals’ breed for the purpose of statistical analyses.

### Phenotypic Correlations

**FIGURE 1:**
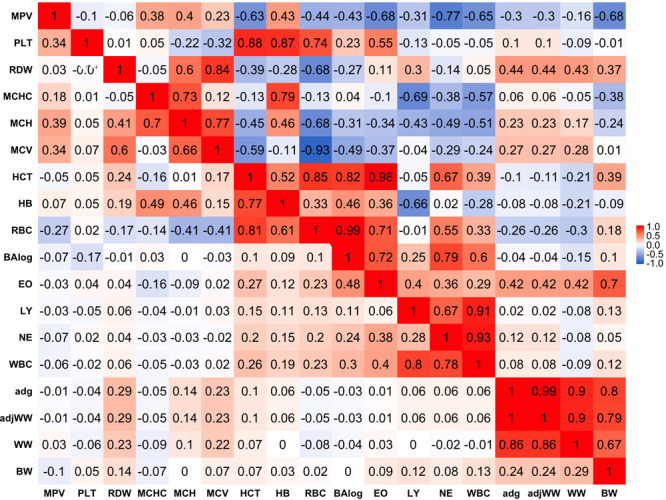
Genetic (above diagonal) and phenotypic (below diagonal) correlations between traits. Traits included are: mean platelet volume (MPV), Platelets (PLT), red blood cell distribution width (RDW), mean corpuscular hemoglobin concentration (MCHC), mean corpuscular hemoglobin (MCH), mean corpuscular volume (MCV), hematocrits (HCT), hemoglobin content(HB), red blood cells (RBC), basophils(log) (Balog), eosinophils (EO), lymphocytes (LY), neutrophils (NE), white blood cells (WBC), average daily gain (ADG), adjusted weaning weight (adjWW), weaning weight (WW) and birth weight (BW). Average daily gain and adjusted weaning weight show a phenotypic correlation of 1 because these traits are a function of each other. Gradient of color from blue to red represents negative to positive correlations and their strength, respectively.

Phenotypic correlations are presented below the diagonal in Figure 1. Stronger phenotypic correlations were observed between similar traits, such as WW and average daily gain (ADG) that had a phenotypic correlation of 0.86. In a similar manner, strong correlations were found between similar blood-based traits. Within red blood cell traits, strong correlations of 0.81 and 0.77 were found between hematocrit percentage (HCT), RBC and hemoglobin content (HB), respectively. Likewise, white blood cell traits tended to show stronger correlations within themselves as in the case of WBC with LY and NE having correlations of 0.80 and 0.78, respectively.

Phenotypic correlations between blood-based and production traits tended to be weak with some exceptions. The strongest negative phenotypic correlation was found between MCHC and WW that showed a correlation of −0.09. ADG and adjusted weaning weight to 205 days (adjWW) showed the strongest positive phenotypic correlation between production traits and blood-based traits, where the correlations with red blood cell distribution width (RDW) were 0.29 for both productivity traits. It should be highlighted that the correlation between ADG and adjWW was 1. Further, WW, ADG and adjWW had highly positive correlations. Both of these findings were expected given that these three traits are directly related as functions using the same weights for their calculations.

### Genetic Correlations

Genetic correlations between traits above the diagonal are shown in Figure 1. Genetic correlations were found to be markedly stronger than phenotypic correlations but followed the same trend of being stronger within trait groups. Birth weight (BW) was the production trait that showed the strongest genetic correlations with blood-based traits, having correlations of -0.68 and 0.70 with mean platelet volume (MPV) and EO, respectively. It should be noted that RDW and EO showed moderate to strong genetic correlations with all four production traits included in the present analyses as shown above the diagonal in Figure 1. Genetic correlations between production traits were strong, where the weakest correlation was 0.80 between BW and ADG.

When evaluating the genetic correlations between blood-based traits, interestingly mean platelet MPV showed moderate to strong correlations with all white blood cell traits included in the analyses (BAlog = −0.43, EO = −0.68, LY = −0.31, NE = −0.77, WBC = −0.65). Mean platelet volume also showed moderate to strong correlations, but predominantly positive ones with red blood cell traits. Platelets (PLT) showed strong genetic correlations with RBC, HB and HCT while intriguingly, showing a weak genetic correlation of −0.1 with MPV.

As noted before with phenotypic correlations, red blood cell traits tended to show strong genetic correlations among themselves. Several red blood cell traits had relatively strong correlations with white blood cell traits. It is worth highlighting the strong positive correlations between HCT and EO with basophils (Balog) and RBC, which were 0.98 and 0.99 respectively. Strong negative correlations were also observed between MCHC and HB with LY of −0.69 and −0.66, respectively.

Strong genetic correlations were found among all white blood cell traits and no negative genetic correlations were found amongst them. WBC had genetic correlations of 0.86 with LY and 0.93 with NE, while BAlog had correlations of 0.72 and 0.79 with NE and EO. Finally, genetic correlations between production traits and blood-based traits ranged from weak to strong with the weakest correlation being between MCV and BW (0.01) and the strongest one (0.70) between BW and EO.

### Estimation of Narrow Sense Heritability (h^2^)

Estimates produced by both Bayesian and frequentist analyses are shown in Table 3. Narrow sense heritability estimates were similar between estimation techniques but estimates from Bayesian approach tended to be lower. The greatest difference between methods was for monocytes (MO). Estimates from the frequentist approach ranged from 0.01 ± 0.05 for MO to 0.60 ± 0.10 for WW, while estimates from Bayesian analyses ranged from 0.11 ± 0.04 to 0.55 ± 0.07 for MO and weaning weight, respectively.

**TABLE 3:** Narrow sense heritability (h^2^) estimates for blood and growth traits.

**Trait**	**Approach**	
	**Bayesian**	**Frequentist**
Average daily gain. (ADG)	0.48 (0.07) ^1^	0.54 (0.11) ^2^
Adjusted weaning weight (adjWW)	0.48 (0.07)	0.54 (0.11)
Birth weight (BW)	0.38 (0.08)	0.41 (0.10)
Weaning weight (WW)	0.55 (0.07)	0.60 (0.10)
Basophils (Balog)	0.15 (0.06)	0.23 (0.10)
Eosinophils (EO)	0.15 (0.05)	0.11 (0.08)
Hemoglobin (HB)	0.25 (0.08)	0.27 (0.10)
Hematocrits (HCT)	0.17 (0.06)	0.11 (0.08)
Lymphocytes (LY)	0.22 (0.07)	0.26 (0.10)
Mean corpuscular hemoglobin (MCH)	0.48 (0.09)	0.52 (0.11)
Mean corpuscular hemoglobin concentration (MCHC)	0.42 (0.09)	0.46 (0.11)
Mean corpuscular volume (MCV)	0.40 (0.09)	0.44 (0.11)
Monocytes (MO)	0.11 (0.04)	0.01 (0.05)
Mean platelet volume (MPV)	0.23 (0.07)	0.24 (0.10
Neutrophils (NE)	0.28 (0.07)	0.30 (0.10)
Platelets (PLT)	0.18 (0.06)	0.16 (0.09)
Red blood cells number (RBC)	0.32 (0.08)	0.35 (0.10)
Red blood cell distribution width(RDW)	0.24 (0.08)	0.18 (0.10)
White blood cells (WBC)	0.31 (0.08)	0.32 (0.10)

*^1^ Sampling error shown in parenthesis. ^2^ Standard error shown in parenthesis.*

It is important to note that while heritability estimates from the Bayesian analyses tended to be lower than those from frequentist analyses, there were cases where Bayesian estimates were larger than frequentist estimates as is the cases of EO, HCT, MO, PLT and RDW. However, only MO shows heritability estimates that are different when standard errors and sampling errors are taken in consideration.

### Genome-Wide Association Study (GWAS)

**FIGURE 2:**
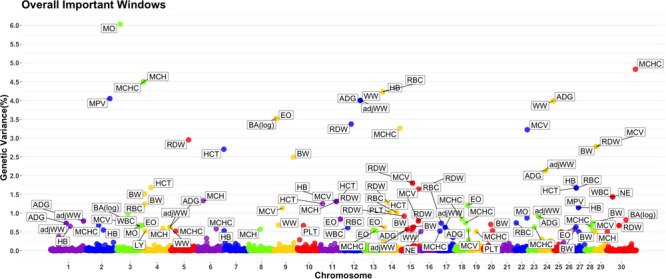
Manhattan plot displaying one-megabase windows and the percentage of estimated genetic variance they account for along the genome. Labeled windows explain ≥ 0.5% of estimated genetic variance. Labels represent the abbreviation of the trait for which the variance is explain at each window. Traits included are: mean platelet volume (MPV), Platelets (PLT), red blood cell distribution width (RDW), mean corpuscular hemoglobin concentration (MCHC), mean corpuscular hemoglobin (MCH), mean corpuscular volume (MCV), hematocrits (HCT), hemoglobin content (HB), red blood cells (RBC), basophils(log) (Balog), eosinophils (EO), lymphocytes (LY), neutrophils (NE), white blood cells (WBC), average daily gain (ADG), adjusted weaning weight (adjWW), weaning weight (WW) and birth weight (BW). Chromosome X is identified as chromosome 30.

A genome-wide association study was performed for each of the nineteen traits included in this study. Overall, 91 one-megabase windows explained more than 0.5% of the estimated genetic variance for at least one trait. All the windows identified are presented in Figure 2. Of these windows, only 15 showed a posterior probability of inclusion (PPI) of at least 60%.

**FIGURE 3:**
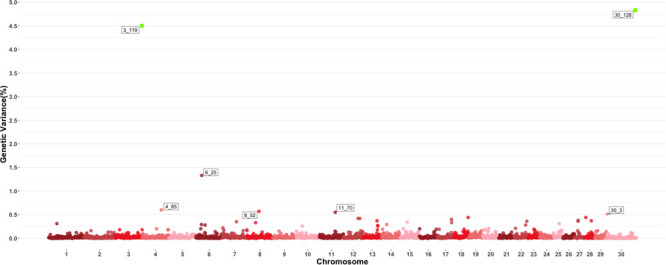
Manhattan plot showing percentage of estimated genetic variance explained by each 1megabase (MB) window for mean corpuscular hemoglobin (MCH). Labeled points explain ≥ 0.5% of the estimated genetic variance. Points highlighted in green have posterior probability of inclusion (PPI) > 60%. The first number of the label of each window represents the chromosome where the window is located, numbers after the underscore. i.e., “6_25” represents a QTL on chromosome 6 encompassing the window from 25–26 Mbs. Chromosome X is identified as chromosome 30.

Figure 3 shows the GWAS results for MCH. For this trait, seven 1-Mb windows were found to be responsible for at least 0.5% of the estimated genetic variance. Windows starting at megabase 119 and 128 on chromosomes 3 and 30 respectively had posterior probabilities larger than 60%, with window 119 on chromosome 3 explaining 4.5% of estimated genetic variance and window 128 on chromosome 30 explaining approximately 4.75%. Several QTL related to average daily gain, meat quality and body conformation were found in these windows along with multiple QTL (Orrù et al., 2012) associated to the trait in close proximity to the window on chromosome 4 identified in this GWAS.

**FIGURE 4:**
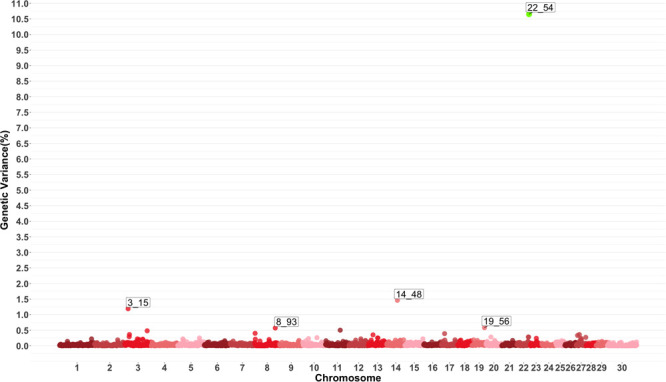
Manhattan plot showing percentage of estimated genetic variance explained by each 1 MB window for monocytes (MO). Labeled points explain ≥ 0.5% of the estimated genetic variance. Points highlighted in green have posterior probability of inclusion (PPI) > 60%. The first number of the label of each window represents the chromosome where the window is located, numbers after the underscore. i.e., “3_15” represents a QTL on chromosome 3 encompassing the window from 15–16 Mbs. Chromosome X is identified as chromosome 30.

Genome-wide association study results for MO are presented in Figure 4 which show five windows explaining greater than 0.5% of the estimated genetic variance located on chromosomes 3, 8, 14, 19 and 22. The window starting at megabase 54 on chromosome 22 had a PPI larger than 60%. There are no previously reported QTL for this trait. Several QTL related to average daily gain, average daily feed intake, body conformation and meat quality were found in the windows described by the GWAS or in close proximity.

**FIGURE 5:**
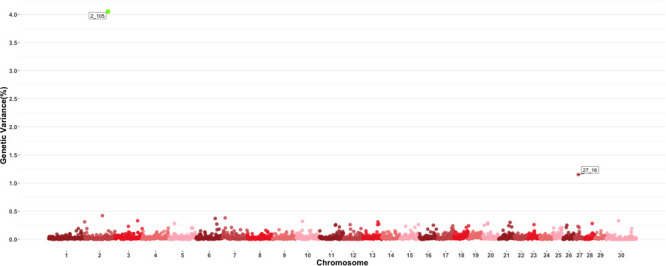
Manhattan plot showing percentage of estimated genetic variance explained by each 1 MB window for mean platelet volume (MPV). Labeled points explain ≥ 0.5% of the estimated genetic variance. Points highlighted in green have posterior probability of inclusion (PPI) > 60%. The first number of the label of each window represents the chromosome where the window is located, numbers after the underscore. i.e., “27_16” represents a QTL on chromosome 27 encompassing the window from 16–17 Mbs. Chromosome X is identified as chromosome 30.

Two windows explaining more than 0.5 of the estimated genetic variance on chromosomes 2 and 27 were found for MPV as shown in Figure 5. The window on chromosome 2 showed a PPI > 60%. There are no QTL related to blood-based traits or growth traits reported in the windows found to be important for this trait, and in a similar fashion to other blood-based traits, there were no QTL previously described to have an effect on MPV values. Given the large number of traits examined in this study, results for all other individual GWAS for all other traits are shown in Supplementary Figures.

Several genomic windows accounted for more than 0.5% of estimated genetic variance for multiple traits. As shown in Table 4, three windows explained greater than 0.5% of the estimated genetic variance for more than two traits. The window that was identified as important for the most traits was at megabase 70 on chromosome 11, which explained approximately 1.25%, 0.6%, 0.7%, 0.7% and 0.5% of HB, HCT, MCH, mean corpuscular volume (MCV) and red blood cell distribution width, respectively. There are no previous reports of QTL associated to HB. All reported QTL for mean corpuscular hemoglobin (MCV), MCH and RDW are found on chromosomes 4, 5, 15 and 25.

**TABLE 4:** Significant windows overlapping over different traits.

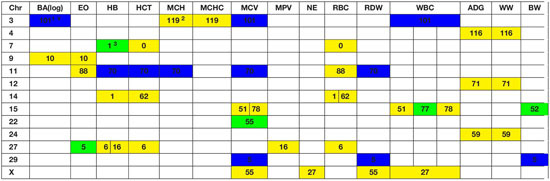

*^1^ Windows highlighted in blue explained more than 0.5% of estimated genetic variance for three or more traits. ^∀^ Numbers in the cell represents the megabase at which the 1-megabase window starts. ^2^ Orange highlighted windows explained more than 0.5% of estimated genetic variance for two traits. ^3^ Green highlighted windows explained more than 0.5% of estimated genetic variance for one trait and are immediately next to a significant window for a different trait.*

### Gene Ontology Enrichment Analysis

Once the significant windows were identified for each trait, gene ontology term enrichment was performed. Overall, the term “unannotated” was significantly enriched for function in 6 traits each for function and process. It is worth highlighting that in the individual trait ontology term enrichment analyses, RDW and BW were both significantly enriched for folic acid receptor activity and binding due to genes *FOLR1*, *FOLR2* and *FOLR3*, spanning over the 51 and 52 Mb on chromosome 15. Additionally, BW was significantly enriched for biological process for terms such as response to oxygen-containing compound and response to endogenous stimulus.

Given the very limited literature and research found in the scientific literature for the specific blood-based traits in cattle examined in this project, the main focus was directed to the broad categories of the traits to increase the chance of finding significant enrichment. Therefore, all the genes identified for traits that fell in the same category (i.e., MCHC and RBC were grouped under red blood cell traits) were grouped into one of four categories before analysis. The categories included red blood cell traits, white blood cell traits, platelet traits and growth traits. In total, 615, 365, 324 and 91 genes were found in windows significant for red blood cell traits, white blood cell traits, growth traits and platelet traits respectively. The 10 most-significantly enriched for each trait category are shown in Table 5. Interestingly, red and white blood cell traits shared significant enrichment for six traits including nitrogen compound metabolic process (FDR ≤ 5%). On the other hand, growth traits shared only one significantly enriched category with platelet traits and two with white blood cell traits while not sharing any with red blood cell traits. Finally, the ten most significant terms that were enriched for biological function for each of the trait categories along with the respective FDR are presented in Table 6. Overall, there was notably less enrichment for biological function, to the point where “unannotated” (FDR < 0.01%) was the only term significantly enriched for traits related to platelets while protein binding (FDR < 0.001% for all categories) was shared by all other categories. As seen with BW, folic acid receptor activity was significantly enriched for growth traits. White blood cell traits showed enrichment for transcription regulatory region DNA binding and regulatory region nucleic acid binding.

**TABLE 5:** The ten most significantly enriched terms for biological process for each trait category.

**Red Blood Cell Traits**	**FDR ^1^**	**Platelet Traits**	**FDR**
cellular process ^2^	0.00%	response to stimulus	4.00%
organic substance metabolic process	0.00%	detection of chemical stimulus involved in sensory perception of smell	3.00%
metabolic process	0.00%	detection of chemical stimulus involved in sensory perception	2.00%
cellular metabolic process	0.00%	sensory perception of smell	2.50%
primary metabolic process	0.00%	detection of chemical stimulus	2.00%
macromolecule metabolic process	0.00%	detection of stimulus involved in sensory perception	2.00%
nitrogen compound metabolic process	0.00%	smooth muscle cell migration	2.00%
cellular component organization or biogenesis	0.00%	sensory perception of chemical stimulus	1.75%
cellular component organization	0.00%	response to chemical	1.56%
macromolecule modification	0.00%	detection of stimulus	1.80%

**White Blood Cell Traits**		**Growth traits**	

organic substance metabolic process	0.00%	cellular process	0.00%
primary metabolic process	0.00%	regulation of biological process	0.00%
nitrogen compound metabolic process	0.00%	cell communication	0.00%
metabolic process	0.00%	biological regulation	0.00%
macromolecule metabolic process	0.00%	regulation of cellular process	0.00%
cellular process	0.00%	signaling	0.00%
cellular metabolic process	0.00%	response to stimulus	0.00%
Localization	0.00%	regulation of cell communication	0.00%
biological regulation	0.00%	regulation of signaling	0.00%
organic substance biosynthetic process	0.00%	positive regulation of biological process	0.00%

*^1^ False discovery rate.*

**TABLE 6:** Ten most significantly enriched terms for function for each trait category.

**Red Blood Cell Traits**	**FDR**^∀^	**Platelet Traits**	**FDR**
binding	0.00%	unannotated	0.00%
protein binding	0.00%		
ion binding	0.00%		
catalytic activity	0.00%		
hydrolase activity	0.00%		
heterocyclic compound binding	0.00%		
organic cyclic compound binding	0.00%		
modified amino acid binding	0.00%		
anion binding	0.00%		
-	-		

**White Blood Cell Traits**		**Growth traits**	

serine-type endopeptidase activity	0.00%	binding	0.00%
serine-type peptidase activity	0.00%	folic acid binding	0.00%
serine hydrolase activity	0.00%	protein binding	0.00%
binding	0.00%	insulin receptor binding	0.50%
protein binding	0.00%	amide binding	2.00%
catalytic activity	0.00%	modified amino acid binding	1.67%
hydrolase activity	0.00%	folic acid receptor activity	2.00%
endopeptidase activity	0.00%	voltage-gated sodium channel activity	1.75%
regulatory region nucleic acid binding	0.22%	protein-containing complex binding	1.56%
transcription regulatory region DNA binding	0.20%		

*^∀^ False discovery rate.*

## Discussion

### Most Blood-Based Traits and Growth Traits Are Weakly Correlated

In the present study, genetic correlations among growth and blood-based traits followed the same pattern than phenotypic correlations with the exceptions of the correlations between MPV and BW, and RDW and EO with all growth traits. Clapperton et al. (2008) reported similar results in swine, describing weak and mostly negative phenotypic correlation between several subsets of white blood cells and ADG ranging from −011 to 0.16. The same study found strong genetic correlations between white blood cell related traits and average daily gain that ranged from −0.58 to 0.23. Those results differ from the results in the present study, perhaps because of differences in species. Leach et al. (2013), found weak and mostly negative genetic correlations between white blood cell traits and ADG, supporting the present findings.

Overall, the findings of this study indicate that phenotypic and genetic correlations with a few exceptions tended to be weak between blood cell traits and growth traits. The weak correlations between blood-based traits and growth traits limit the potential for blood-based traits to be used as indicators of performance under varying environments. Strong genetic correlations between blood-based traits indicate the existence of an important overlap in genetic control and can be considered as evidence of pleiotropic effects playing a role in regulating multiple blood- based traits as found previously by Lukowski et al. (2017).

### Blood-Based Traits Tend to Have Moderate to High Heritability

Heritability (h^2^) estimates in the present study are in line with what is generally reported in the literature (Kitchenham and Rowlands, 1976; Bourdon and Brinks, 1982; Rowlands et al., 1983; Arnold et al., 1991; Bullock et al., 1993; Bennett and Gregory, 1996; Phocas and Laloë, 2004; Wright et al., 2014; Mpetile et al., 2015; Snelling et al., 2019). Heritability estimates (h^2^) for white blood cell traits by Leach et al. (2013) ranged from 0.28 to 0.50, confirming the findings of the present study, with the only exception of MO which had a heritability estimate (h^2^) of 0.11 and 0.01 for Bayesian and frequentist approaches respectively, which in both cases are lower than the estimates previously reported by Leach et al. (2013) that ranged from 0.21 to 0.39. After an extensive literature review, we believe the present findings provide one of the few, if not the genetic parameter estimates for several blood-based traits in beef cattle at weaning age.

Bayesian analyses tended to produce lower estimates of heritability than frequentist analyses, indicating the possibility of missing heritability. Missing rare variants is possible in this case given that the population used to produce the estimates is relatively small (∼570 animals) and thus, a larger study population might be needed to capture rare variants. However, the GGP F250 SNPchip is the chip with the highest number of rare variants included and therefore should capture all the rare variants present in our population. To differentiate between missing heritability and the possibility of missing genetic variance because the pi value used in the Bayesian analyses was too large and therefore not taking in consideration all markers that explained genetic variance, heritability estimates (data not shown) were produced using Bayes C priors with a pi value of zero. The difference in estimates of heritability when using the different pi values was minimal. Possible reasons for missing heritability may be the small sample size, epistatic interactions between markers, other structural variations in the genome like copy number variants (CNV), linkage disequilibrium (LD) or rare alleles not present in the studied population (Clarke and Cooper, 2010; Vineis and Pearce, 2010; Makowsky et al., 2011; Zuk et al., 2012). Other possible explanations for the missing genetic variance could be that the SNP chips used to genotype the animals did not include markers that explain variance for the trait, or perhaps some of the rare variants that explained genetic variance for the traits were lost through filtering SNPs that had MAF lower than 0.02. Another possible cause could be the different marker information content given the different allele frequencies in the breeds that are admixed in the population used for this research. However, the Bovine GGP F250 SNPchip contains a very large number of rare variants and includes data from most of the breeds that compose the population used for this study (NEOGEN, 2016).

### Maternal Genetic Effects Do Not Impact Genetic and Genomic Correlations or Heritability

Another concern was that heritability estimates could be inflated due to maternal genetic effects. Given that blood-based and productivity traits were measured at weaning and that the original analyses did not take in consideration maternal effects that might be significant, a model accounting for maternal effects was implemented using ASReml (Mary et al., 2009) (data not shown). The difference in estimates from a model with and without genetic maternal effects was very small, indicating that maternal effects have limited influence on blood-based traits and productivity at weaning. However, estimation of maternal effects is complex and given the amount of data available these results should be taken as preliminary.

### Genome Wide Association Study Results Identify Few Genomic Regions With Large Effects

Although the GWAS performed for each trait revealed 91 windows of 1-megabase in length that explained at least 0.5% of the estimated genetic variance, the most interesting results were the numerous overlaps of windows that were important for different traits. Windows of importance were identified on chromosome 23 only for three traits, ADG, adjWW and MCHC. No GWAS windows for any blood-based trait associated to white blood cells on chromosome 23 were identified, where the MHC complex is found in bovines (Steiner et al., 2014). These results provide evidence of possible pleiotropic genes influencing multiple traits related to blood-based traits as well as influencing blood-based traits and productivity traits.

### Several Candidate Genes Were Identified for Windows Associated With Blood-Based Traits and Overlapping With Growth Traits

The only overlap found in white blood cell traits is found on chromosome 9, megabase 10. This window contains genes like *SMAP1*, a protein-coding gene that has been linked to erythropoietic and overall hematopoietic activity in mice (Behl et al., 2012) and receptor endocytosis in mammals (Sato et al., 1998), which makes it a good candidate gene for further studies.

In the case of red blood cell traits, the most important window was found on chromosome 11, starting at position 70 megabases. This window was significant for five traits. A total of 20 annotated genes were identified in this window. Promising candidate genes included *CAPN13*, a gene previously associated to hypertension in humans (Kobayashi et al., 2014) and *LCLAT1*, previously identified to control development of hematopoietic and endothelial lineages in mice embryos (Taylor et al., 2010). Other interesting candidate genes for further research were *YPEL5* and *SPDYA*, that have been linked to cellular cycle progression (Wang et al., 2007) and regulation of CD133 + cell population in humans (Hosono et al., 2010).

Another genomic region with overlapping associations across multiple traits was found at 5-6Mb on chromosome 29. This window overlapped over traits associated to blood cells (MCV and RDW) and performance (BW). A total of ten annotated genes and 18 unannotated genes were found in this region, with no good candidates identified. A set of windows located at 51-53 Mb on chromosome 15 overlapped over these same traits. As shown in the results section, genes associated to folic acid binding (*FOLR1*, *FOLR2* and *FOLR3*). In cattle, it has been shown that folate supplementation can reduce the occurrence of dystocia by up to 50% (Duplessis et al., 2014).

It has also been shown that folate can increase milk production and can modify concentration of amin acids in blood plasma (Graulet et al., 2007). In humans, it has been shown that folate plays a crucial role in nucleic acid synthesis, cell division, regulation of gene expression, amino acid metabolism and neurotransmitter synthesis during fetal development (Djukic, 2007). More importantly, during pregnancy folate intake is crucial for rapid cell proliferation and tissue growth in the uterus and placenta, growth of the fetus and expansion of maternal blood flow (Rondo and Tomkins, 2000) to the point that in humans, folate requirements are 5 to 10 fold greater during pregnancy (Antony, 2007). Although in humans, significantly increased birth weight has been observed when women take folic acid supplementation during gestation (Fekete et al., 2012), no effect has been found in cattle (Girard et al., 1995). In cattle, there is evidence of folic acid supplementation leading to increased milk production over a complete lactation from cows on their second lactation or greater (Girard et al., 1995) which could be translated into greater weaning weights in calves from cows with optimum folate intake. There is a link between folate and blood-based traits. Previous research has shown that folate is required for proliferation of erythroblasts during differentiation (Koury and Ponka, 2004). Moreover, folate and iron deficiency cause erythroblast apoptosis through the impairment of protein and DNA (Blount and Ames, 1995). Although the candidate genes identified in the different windows have been liked to biological processes related to blood-based traits it is important to keep in mind that these genes have not yet been studied in cattle, and therefore further research in beef cattle is needed to elucidate their roles and their potential use as a tool for breeders to accelerate genetic improvement.

## Conclusion

The present study represents one of the first efforts to identify the genetic basis of blood-based traits in beef cattle. The results presented in this study allow us to conclude that: (1) blood-based traits have weak phenotypic correlations, but strong genetic correlations among themselves compared to growth traits. (2) Blood-based traits have moderate to high heritability. (3) There is evidence of an important overlap between genetic control among similar blood-based traits and between some blood-based traits and growth traits. Additionally, multiple windows overlapping over blood-based traits and growth traits and candidate genes that show a biological function that ties these traits together were identified.

The present study also provides evidence that most blood-based traits are heritable, with some exhibiting correlations with growth traits.

Further studies are warranted to determine if CBCs may act as indicators of growth performance under different environments as a means of capturing relationships with immune status, nutrition and environment under different production settings.

## Data Availability Statement

The datasets generated for this study can be found in the Open Science Framework: doi: 10.17605/OSF.IO/E4QMU.

## Ethics Statement

The animal study was reviewed and approved by University of Arkansas Institute for Animal Care and Use Committee (IACUC), protocol number 16037.

## Author Contributions

JK did the experimental design. DH, JD, JP, and EB performed the livestock management and data collection. JC-V, LK, MR, KA, and JK did the data analysis and management. JC-V, LK, MR, KS, and JK wrote the manuscript. MR, KS, LK, and JK did the manuscript review and editing. All authors contributed to the article and approved the submitted version.

## Conflict of Interest

The authors declare that the research was conducted in the absence of any commercial or financial relationships that could be construed as a potential conflict of interest.

## Footnotes

1 https://www.ncbi.nlm.nih.gov/genome/gdv/browser/genome/?id=GCF_002263795.1

2 http://useast.ensembl.org/biomart/martview/
