# Supplementary material for: Genetic Basis of Blood-Based Traits and Their Relationship With Performance and Environment in Beef Cattle at Weaning
Source: Front Genet. 2020 Jul 3;11:717. doi: 10.3389/fgene.2020.00717 (PMC7350949; doi:10.3389/fgene.2020.00717)
Supplement: Supplementary file 1 [file Table_1.DOCX]

**Supplemental Figure 1.** Manhattan plot showing percentage of estimated genetic variance explained by each 1MB window for average daily gain (ADG).

Labeled points explain ≥ 0.5% of the estimated genetic variance. Points highlighted in green have posterior probability of inclusion (PPI) > 60%. The first number of the label of each window represents the chromosome where the window is located, numbers after the underscore indicate the mega base number where the window starts.

**Supplemental Figure 2.** Manhattan plot showing percentage of estimated genetic variance explained by each 1MB window for adjusted weaning weight (adjWW).

Labeled points explain ≥ 0.5% of the estimated genetic variance. Points highlighted in green have posterior probability of inclusion (PPI) > 60%. The first number of the label of each window represents the chromosome where the window is located, numbers after the underscore indicate the mega base number where the window starts.

**Supplemental Figure 3.** Manhattan plot showing percentage of estimated genetic variance explained by each 1MB window for weaning weight (WW).

Labeled points explain ≥ 0.5% of the estimated genetic variance. Points highlighted in green have posterior probability of inclusion (PPI) > 60%. The first number of the label of each window represents the chromosome where the window is located, numbers after the underscore indicate the mega base number where the window starts.

**Supplemental Figure 4.** Manhattan plot showing percentage of estimated genetic variance explained by each 1MB window for birth weight (BW).

Labeled points explain ≥ 0.5% of the estimated genetic variance. Points highlighted in green have posterior probability of inclusion (PPI) > 60%. The first number of the label of each window represents the chromosome where the window is located, numbers after the underscore indicate the mega base number where the window starts.

**Supplemental Figure 5.** Manhattan plot showing percentage of estimated genetic variance explained by each 1MB window for basophils (BAlog).

Labeled points explain ≥ 0.5% of the estimated genetic variance. Points highlighted in green have posterior probability of inclusion (PPI) > 60%. The first number of the label of each window represents the chromosome where the window is located, numbers after the underscore indicate the mega base number where the window starts.

**Supplemental Figure 6.** Manhattan plot showing percentage of estimated genetic variance explained by each 1MB window for eosinophils (EO).

Labeled points explain ≥ 0.5% of the estimated genetic variance. Points highlighted in green have posterior probability of inclusion (PPI) > 60%. The first number of the label of each window represents the chromosome where the window is located, numbers after the underscore indicate the mega base number where the window starts.

**Supplemental Figure 7.** Manhattan plot showing percentage of estimated genetic variance explained by each 1MB window for lymphocytes (LY).

Labeled points explain ≥ 0.5% of the estimated genetic variance. Points highlighted in green have posterior probability of inclusion (PPI) > 60%. The first number of the label of each window represents the chromosome where the window is located, numbers after the underscore indicate the mega base number where the window starts.

**Supplemental Figure 8.** Manhattan plot showing percentage of estimated genetic variance explained by each 1MB window for neutrophils (NE).

Labeled points explain ≥ 0.5% of the estimated genetic variance. Points highlighted in green have posterior probability of inclusion (PPI) > 60%. The first number of the label of each window represents the chromosome where the window is located, numbers after the underscore indicate the mega base number where the window starts.

**Supplemental Figure 9.** Manhattan plot showing percentage of estimated genetic variance explained by each 1MB window for white blood cells (WBC).

Labeled points explain ≥ 0.5% of the estimated genetic variance. Points highlighted in green have posterior probability of inclusion (PPI) > 60%. The first number of the label of each window represents the chromosome where the window is located, numbers after the underscore indicate the mega base number where the window starts.

**Supplemental Figure 10.** Manhattan plot showing percentage of estimated genetic variance explained by each 1MB window for hemoglobin (HB).

Labeled points explain ≥ 0.5% of the estimated genetic variance. Points highlighted in green have posterior probability of inclusion (PPI) > 60%. The first number of the label of each window represents the chromosome where the window is located, numbers after the underscore indicate the mega base number where the window starts.

**Supplemental Figure 11.** Manhattan plot showing percentage of estimated genetic variance explained by each 1MB window for hematocrits (HCT).

Labeled points explain ≥ 0.5% of the estimated genetic variance. Points highlighted in green have posterior probability of inclusion (PPI) > 60%. The first number of the label of each window represents the chromosome where the window is located, numbers after the underscore indicate the mega base number where the window starts.

**Supplemental Figure 12.** Manhattan plot showing percentage of estimated genetic variance explained by each 1MB window for mean corpuscular hemoglobin concentration (MCHC).

Labeled points explain ≥ 0.5% of the estimated genetic variance. Points highlighted in green have posterior probability of inclusion (PPI) > 60%. The first number of the label of each window represents the chromosome where the window is located, numbers after the underscore indicate the mega base number where the window starts.

**Supplemental Figure 13.** Manhattan plot showing percentage of estimated genetic variance explained by each 1MB window for mean corpuscular volume (MCV).

Labeled points explain ≥ 0.5% of the estimated genetic variance. Points highlighted in green have posterior probability of inclusion (PPI) > 60%. The first number of the label of each window represents the chromosome where the window is located, numbers after the underscore indicate the mega base number where the window starts.

**Supplemental Figure 14.** Manhattan plot showing percentage of estimated genetic variance explained by each 1MB window for red blood cells (RBC).

Labeled points explain ≥ 0.5% of the estimated genetic variance. Points highlighted in green have posterior probability of inclusion (PPI) > 60%. The first number of the label of each window represents the chromosome where the window is located, numbers after the underscore indicate the mega base number where the window starts.

**Supplemental Figure 15.** Manhattan plot showing percentage of estimated genetic variance explained by each 1MB window for red blood cell distribution width (RDW).

Labeled points explain ≥ 0.5% of the estimated genetic variance. Points highlighted in green have posterior probability of inclusion (PPI) > 60%. The first number of the label of each window represents the chromosome where the window is located, numbers after the underscore indicate the mega base number where the window starts.

**Supplemental Figure 16.** Manhattan plot showing percentage of estimated genetic variance explained by each 1MB window for platelets (PLT).

Labeled points explain ≥ 0.5% of the estimated genetic variance. Points highlighted in green have posterior probability of inclusion (PPI) > 60%. The first number of the label of each window represents the chromosome where the window is located, numbers after the underscore indicate the mega base number where the window starts.
