# Supplementary figures and images for: Genetic Basis of Blood-Based Traits and Their Relationship With Performance and Environment in Beef Cattle at Weaning
Source: Front Genet. 2020 Jul 3;11:717. doi: 10.3389/fgene.2020.00717 (PMC7350949; doi:10.3389/fgene.2020.00717)

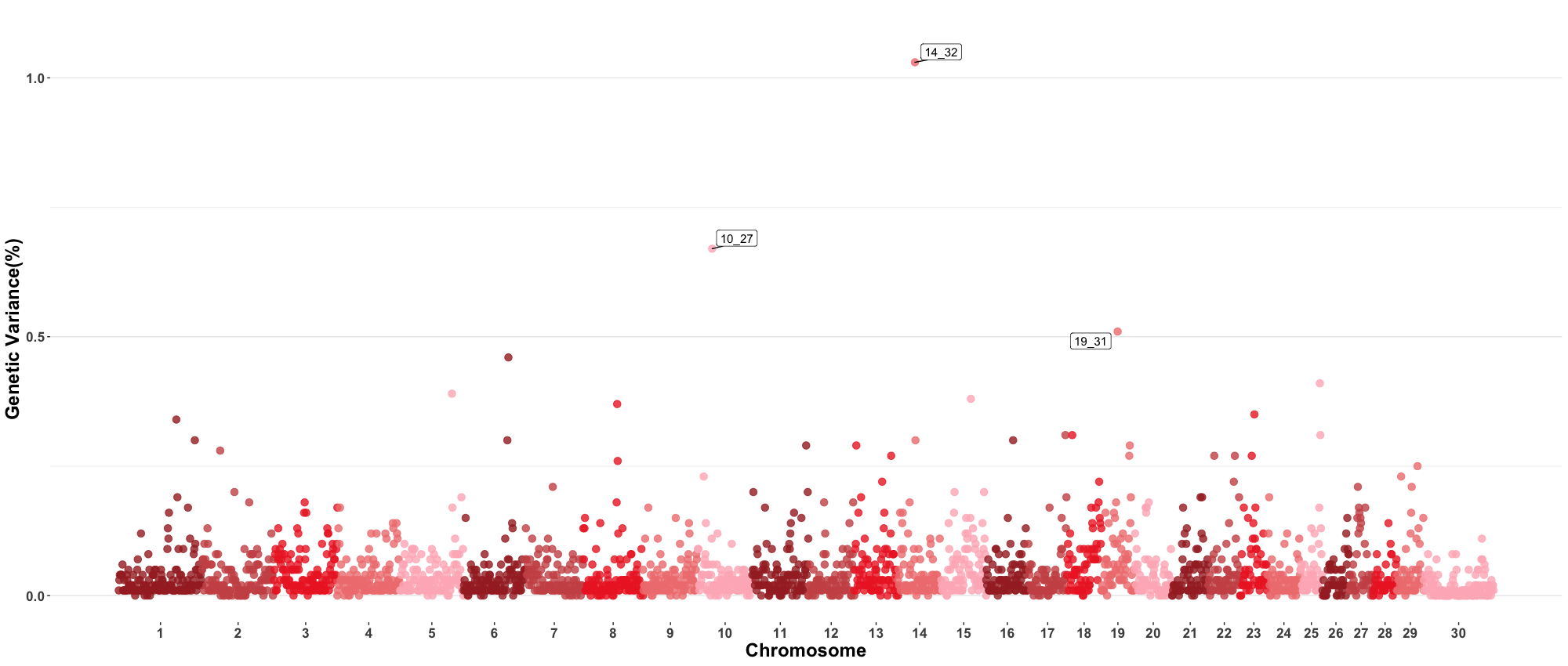

Supplement: Supplementary file 2 [file Data_Sheet_1.ZIP › sup_fig16.tiff]

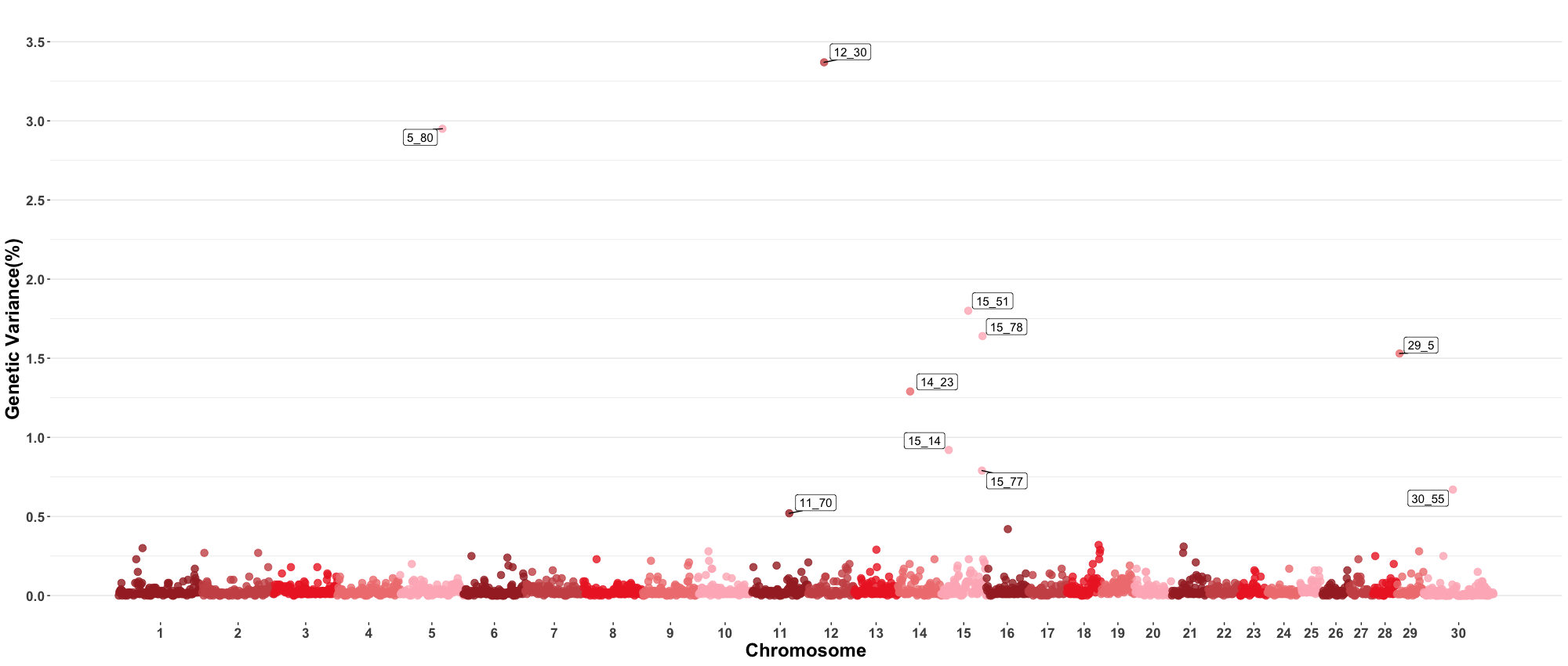

Supplement: Supplementary file 2 [file Data_Sheet_1.ZIP › sup_fig15.tiff]

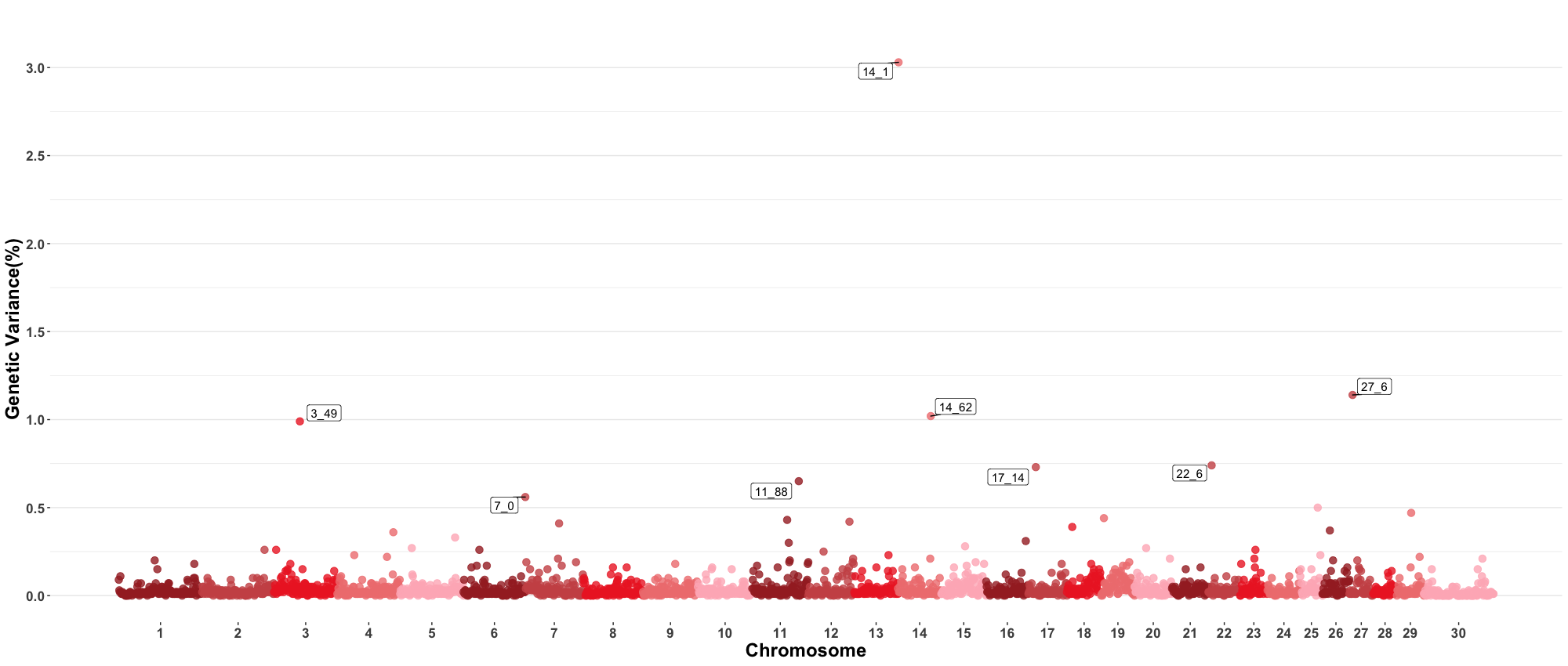

Supplement: Supplementary file 2 [file Data_Sheet_1.ZIP › sup_fig14.tiff]

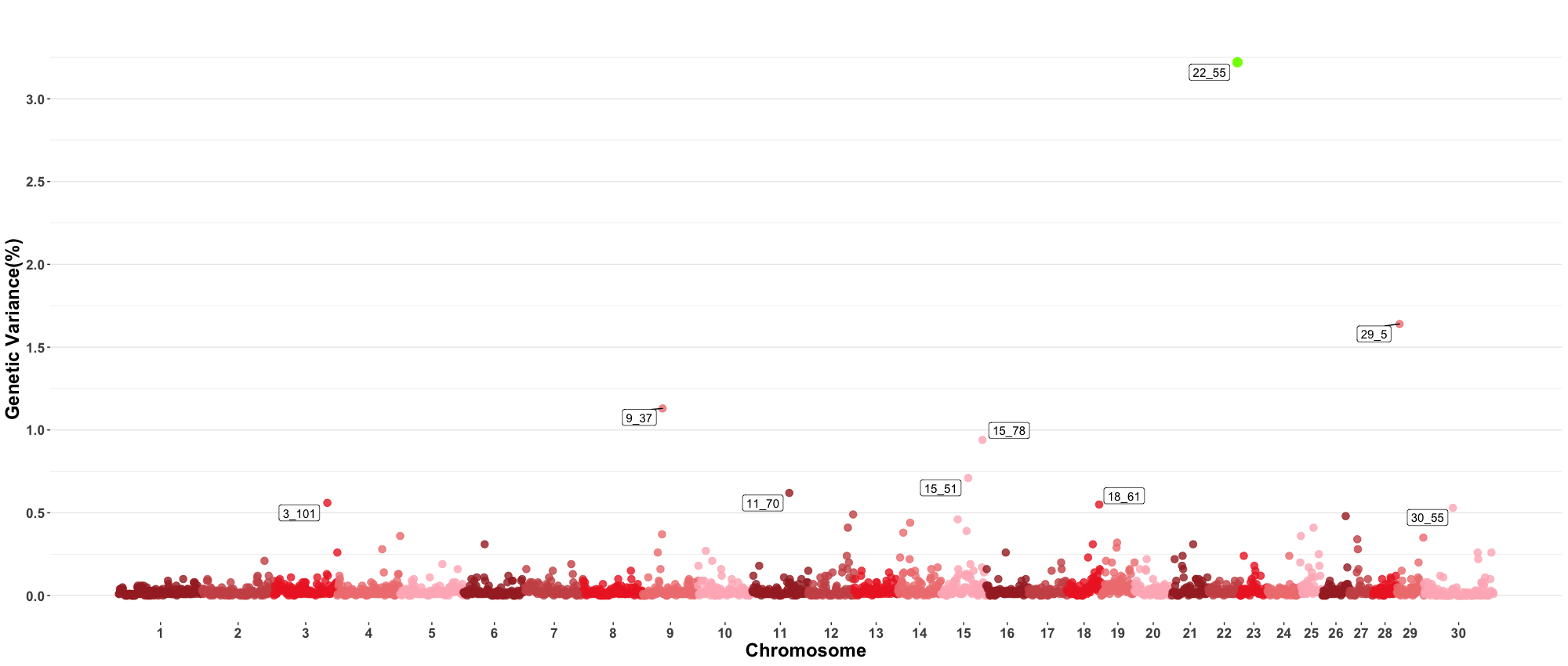

Supplement: Supplementary file 2 [file Data_Sheet_1.ZIP › sup_fig13.tiff]

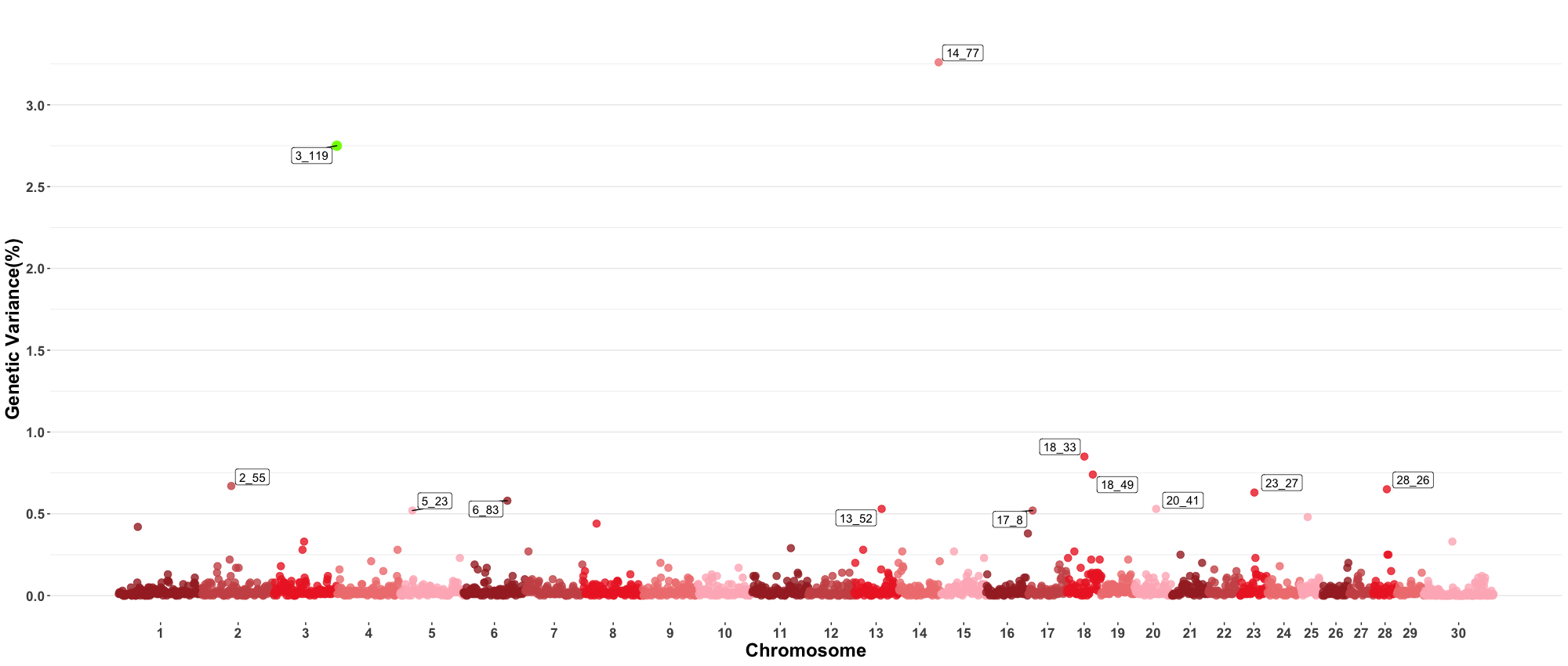

Supplement: Supplementary file 2 [file Data_Sheet_1.ZIP › sup_fig12.tiff]

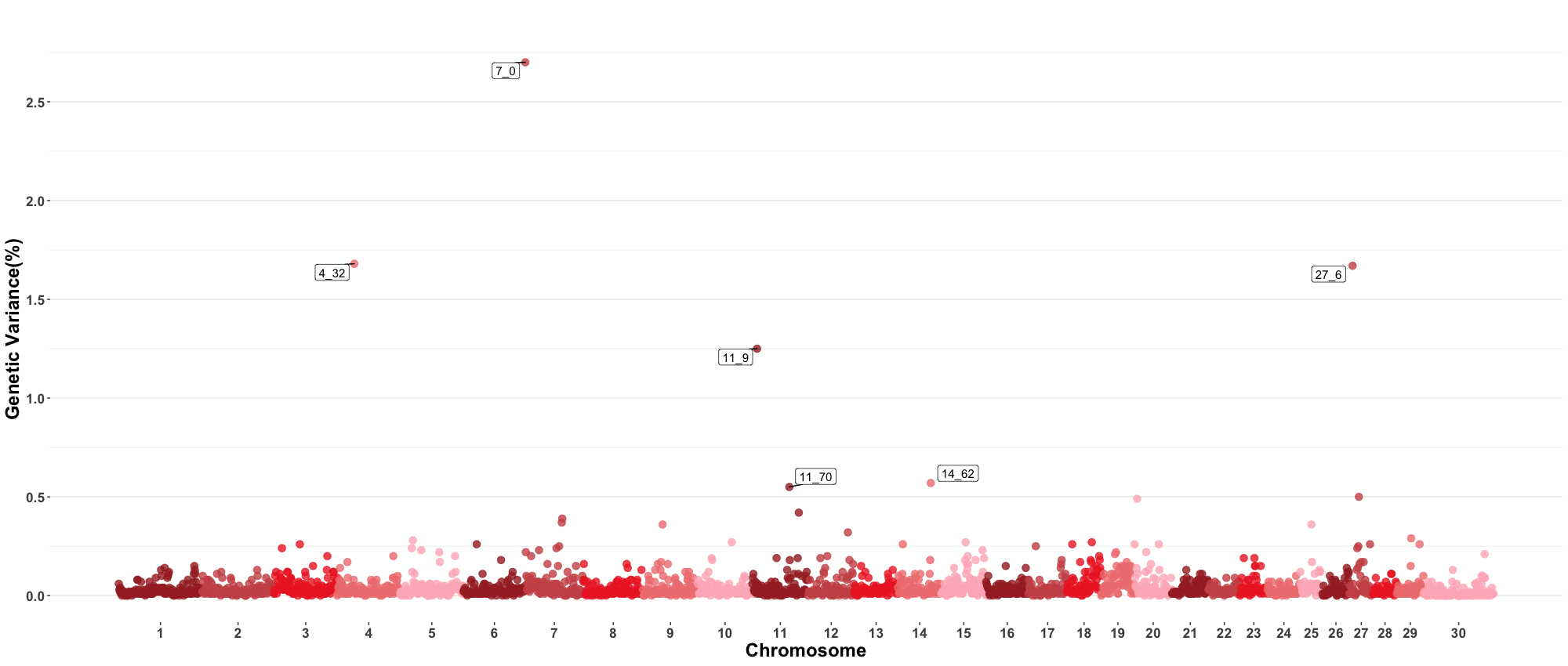

Supplement: Supplementary file 2 [file Data_Sheet_1.ZIP › sup_fig11.tiff]

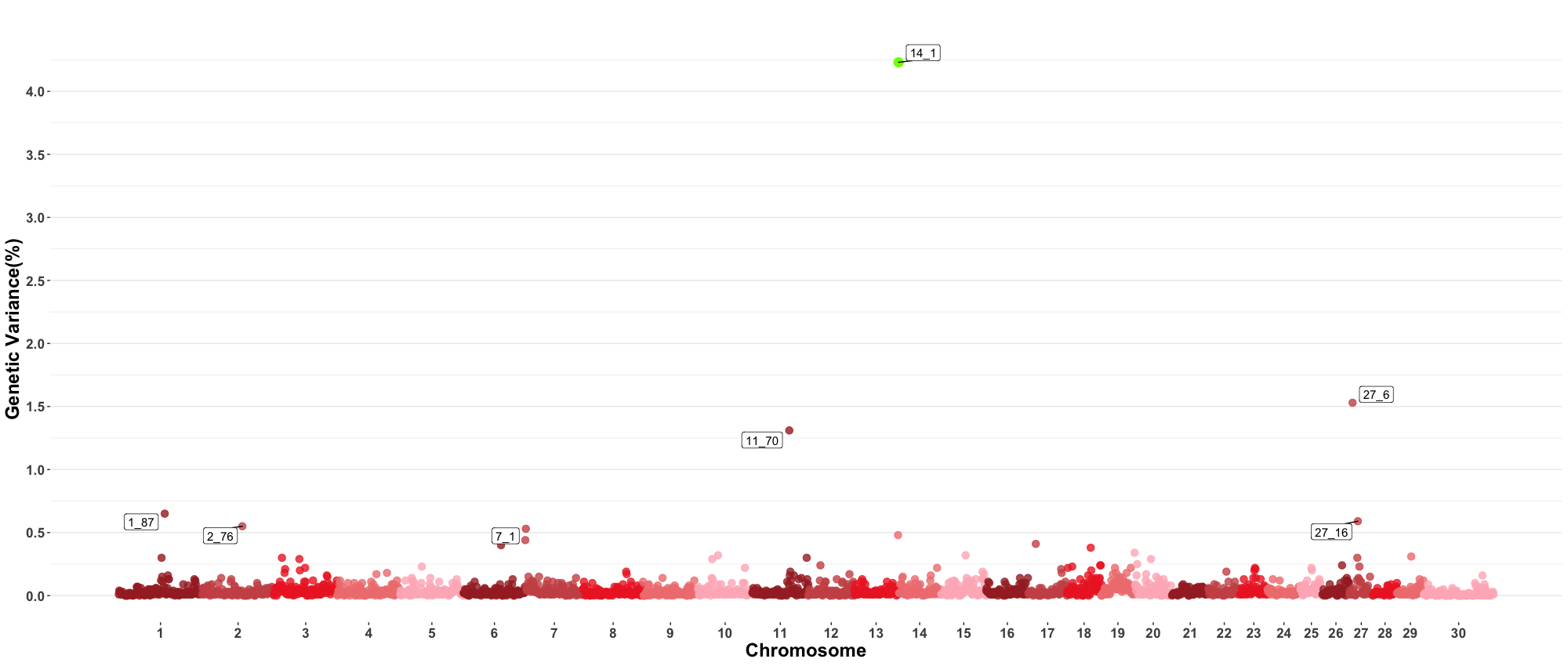

Supplement: Supplementary file 2 [file Data_Sheet_1.ZIP › sup_fig10.tiff]

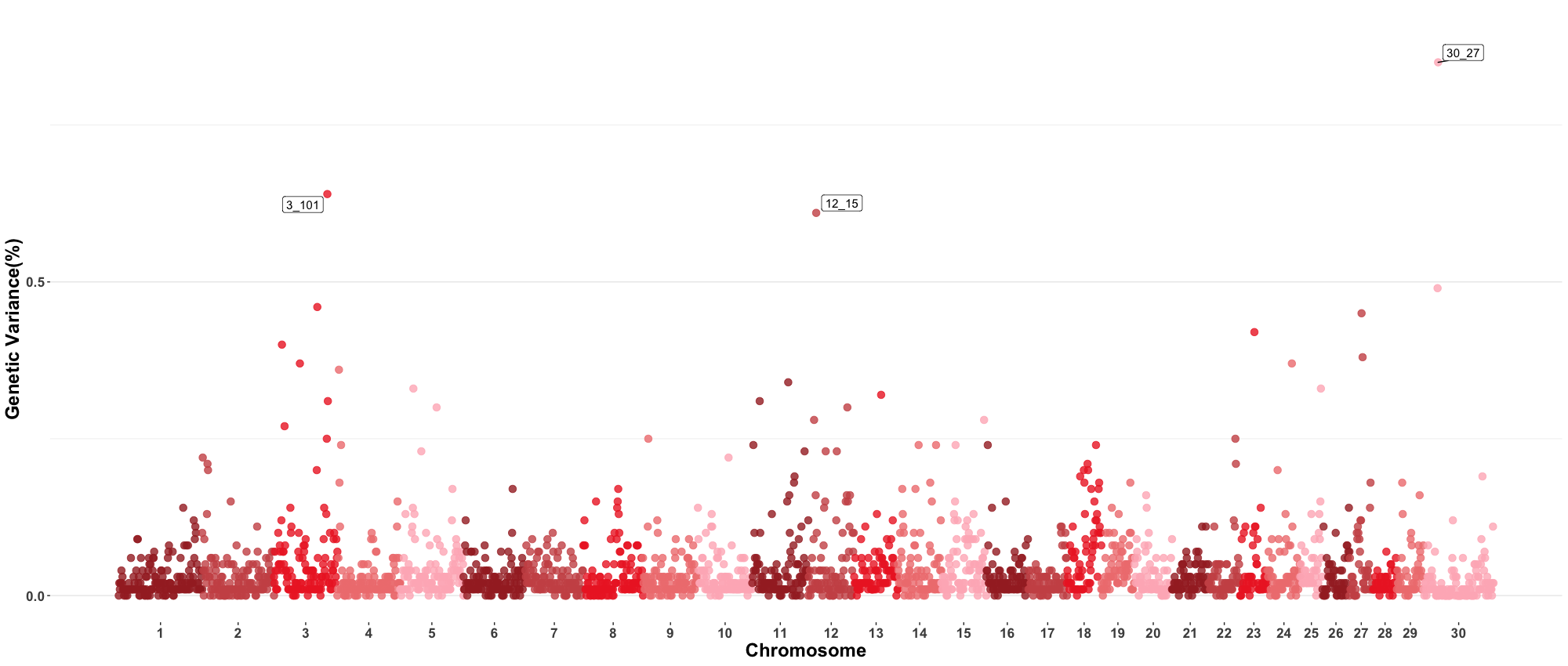

Supplement: Supplementary file 2 [file Data_Sheet_1.ZIP › sup_fig9.tiff]

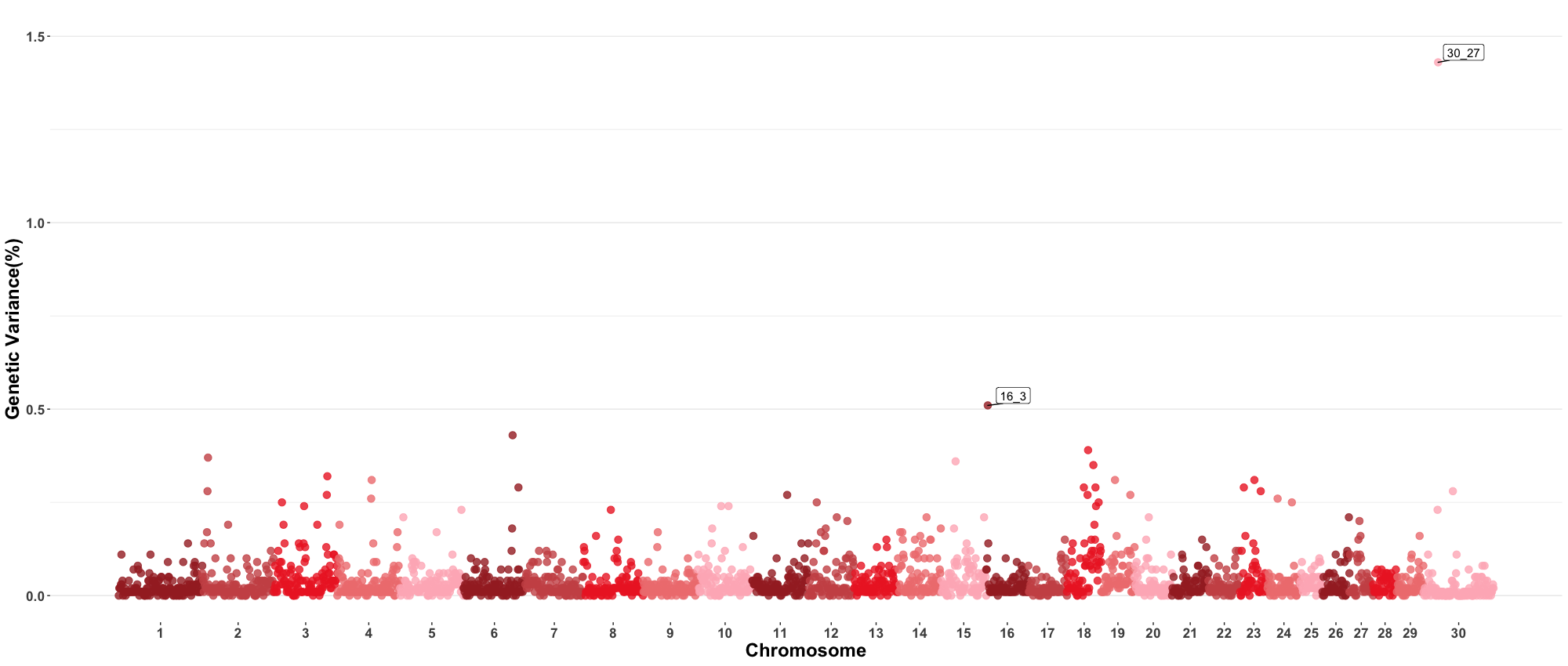

Supplement: Supplementary file 2 [file Data_Sheet_1.ZIP › sup_fig8.tiff]

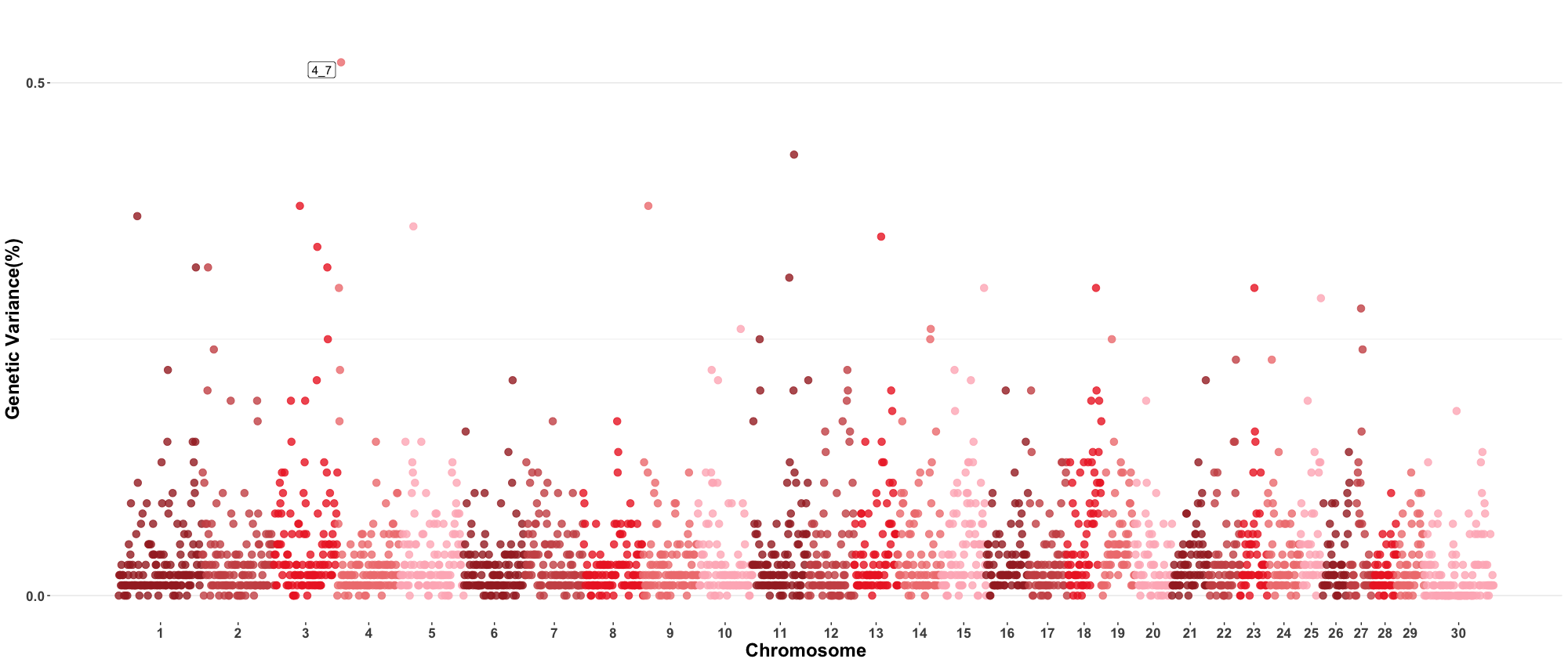

Supplement: Supplementary file 2 [file Data_Sheet_1.ZIP › sup_fig7.tiff]

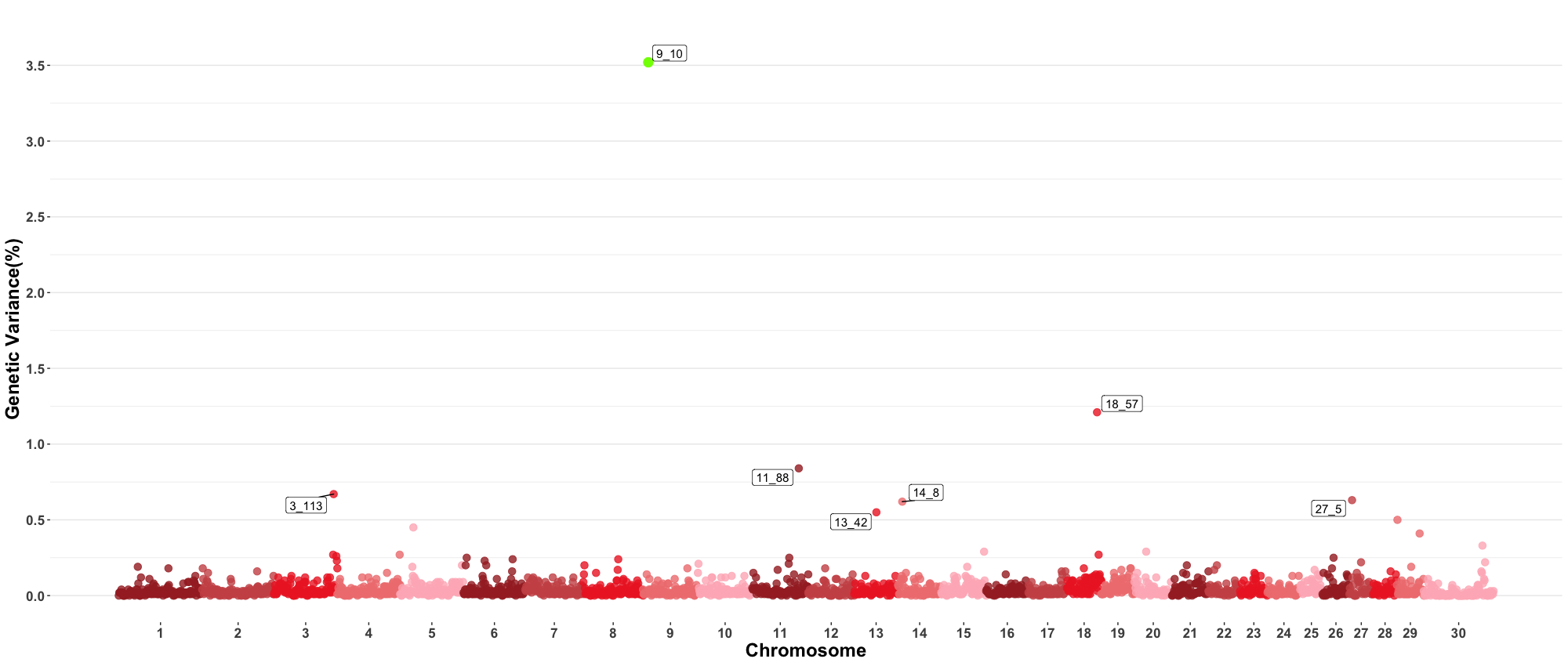

Supplement: Supplementary file 2 [file Data_Sheet_1.ZIP › sup_fig6.tiff]

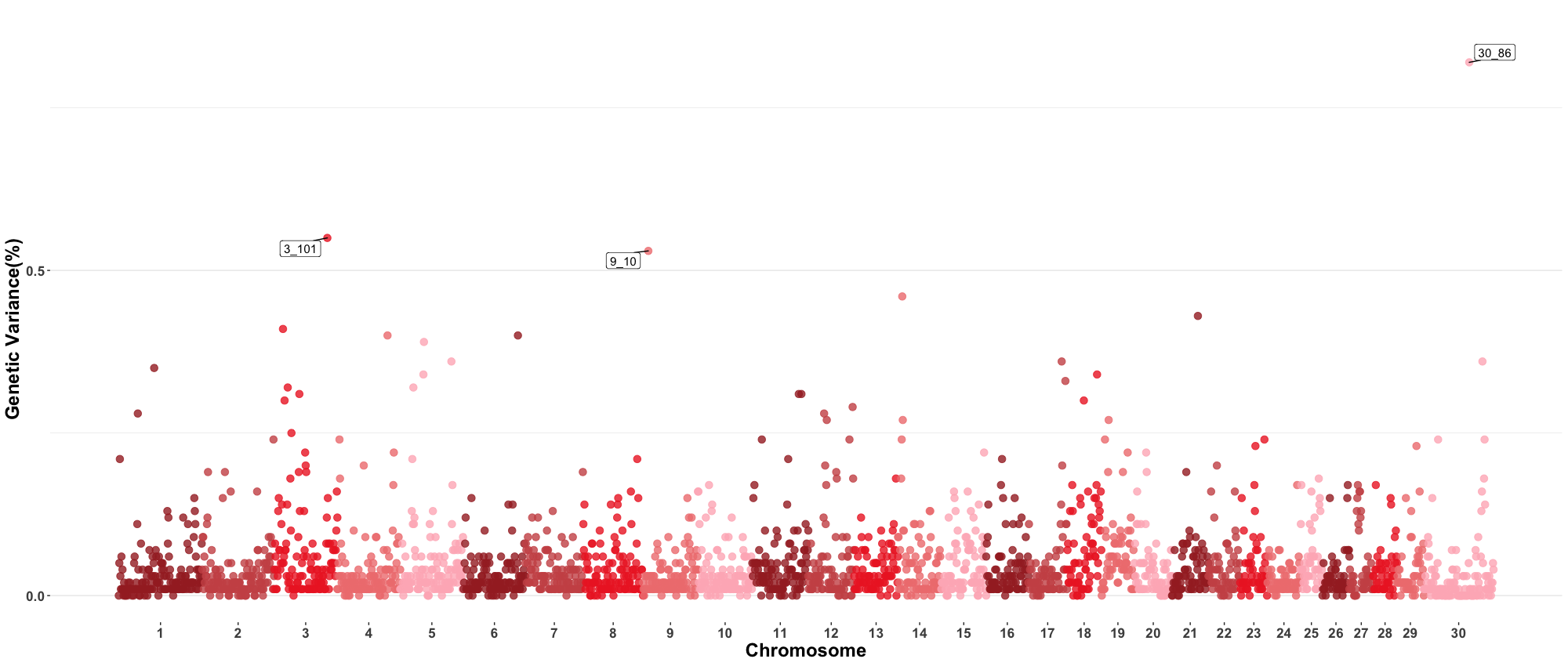

Supplement: Supplementary file 2 [file Data_Sheet_1.ZIP › sup_fig5.tiff]

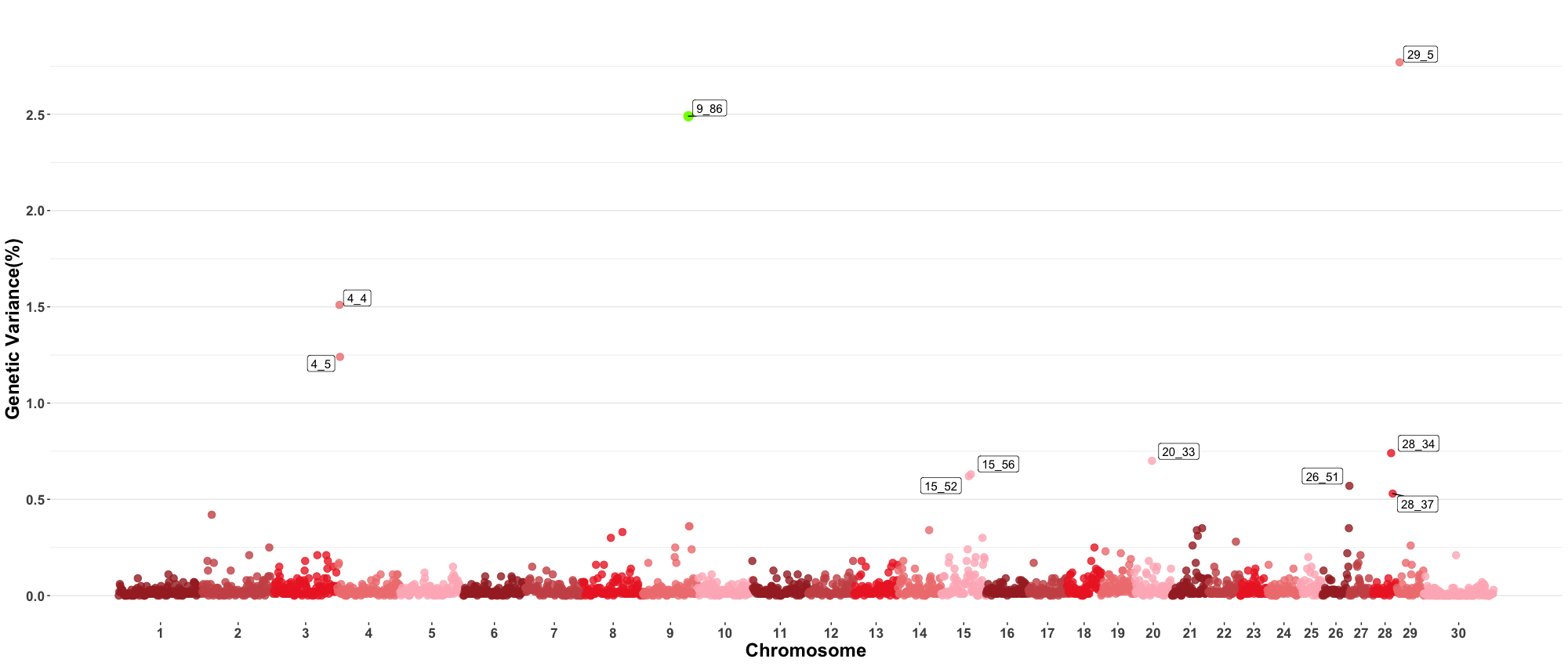

Supplement: Supplementary file 2 [file Data_Sheet_1.ZIP › sup_fig4.tiff]

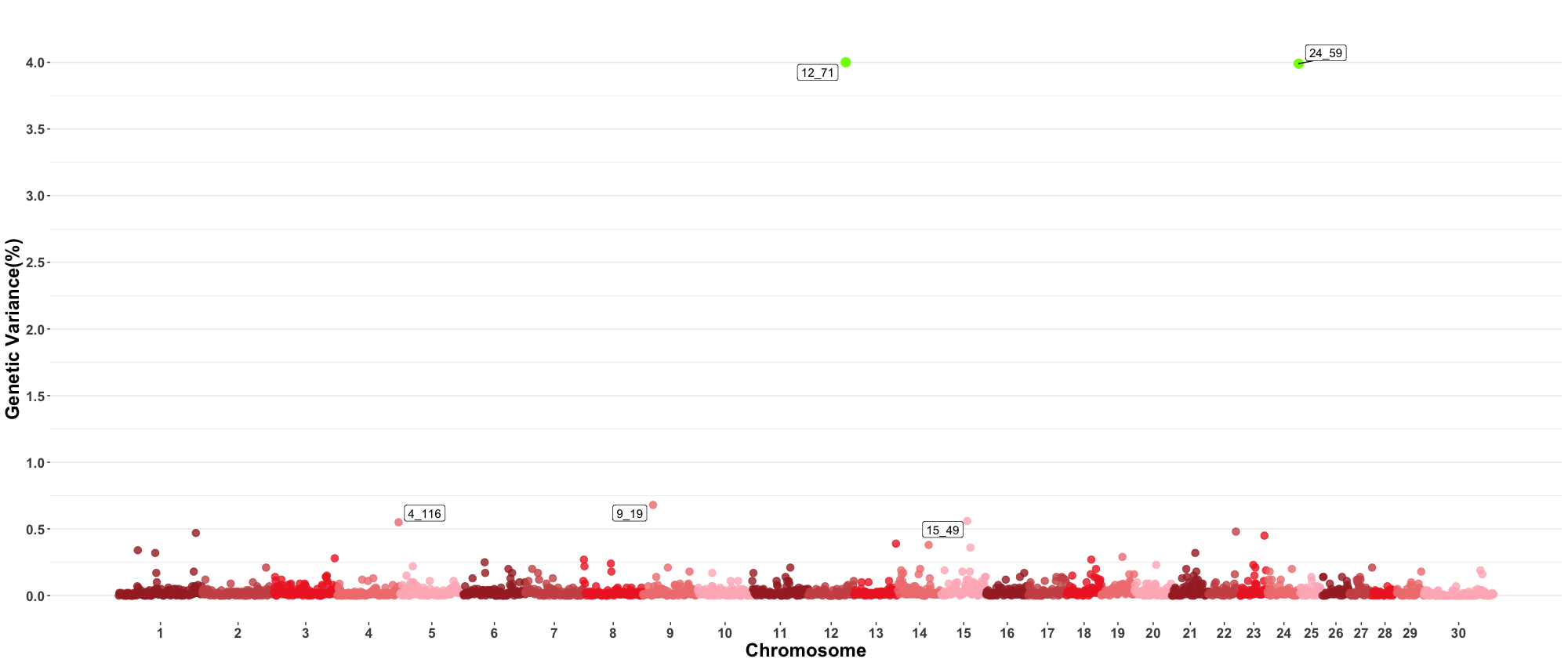

Supplement: Supplementary file 2 [file Data_Sheet_1.ZIP › sup_fig3.tiff]

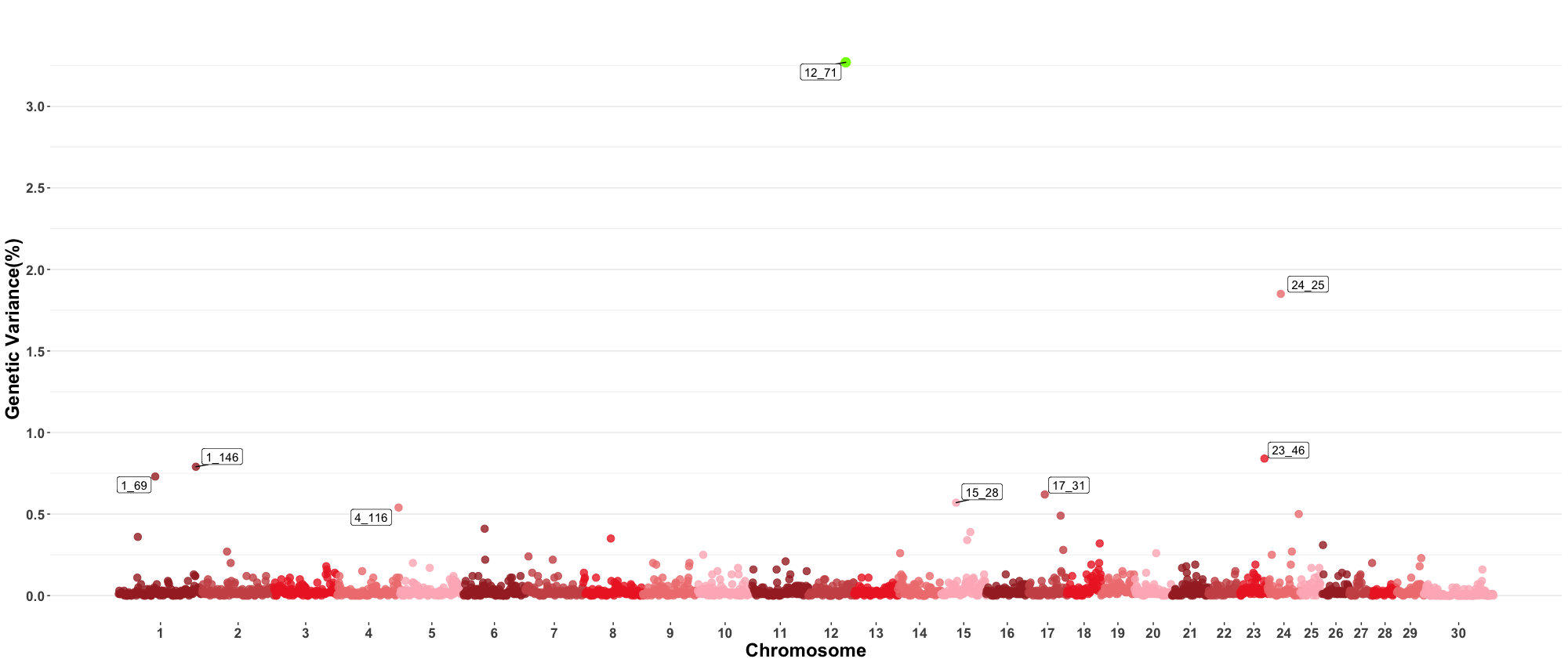

Supplement: Supplementary file 2 [file Data_Sheet_1.ZIP › sup_fig2.tiff]

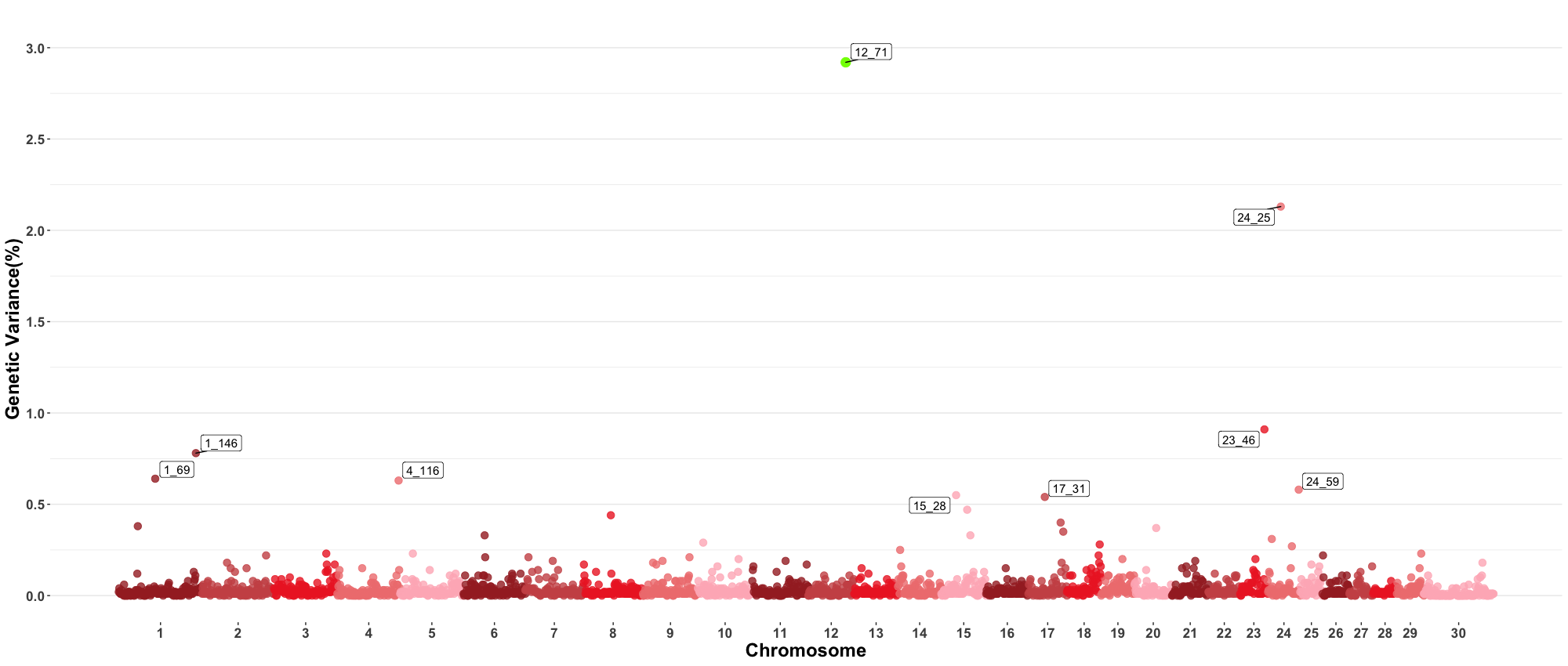

Supplement: Supplementary file 2 [file Data_Sheet_1.ZIP › sup_fig1.tiff]
